# Scoping Review: Innovations in Clinical Neurology Education

**DOI:** 10.1212/NE9.0000000000200048

**Published:** 2023-02-21

**Authors:** William Denney Zimmerman, Melissa B. Pergakis, Emily F. Gorman, Melissa Motta, Peter H. Jin, Rachel Marie E. Salas, Nicholas A. Morris

**Affiliations:** From the Department of Neurology (W.D.Z., M.B.P., M.M., P.H.J., N.A.M.), University of Maryland School of Medicine; Program in Trauma (W.D.Z., M.B.P., M.M., N.A.M.), R Adams Cowley Shock Trauma Hospital; Health Sciences and Human Services Library (E.F.G.), University of Maryland; and Department of Neurology (R.M.E.S.), Johns Hopkins School of Medicine, Baltimore, MD.

## Abstract

Advances in adult learning theory and instructional technologies provide opportunities to improve neurology knowledge acquisition. This scoping review aimed to survey the emerging landscape of educational innovation in clinical neurology. With the assistance of a research librarian, we conducted a literature search on November 4, 2021, using the following databases: PubMed, Embase, Scopus, Cochrane Library, Education Resources Information Center, and PsycINFO. We included studies of innovative teaching methods for medical students through attending physician-level learners and excluded interventions for undergraduate students and established methods of teaching, as well as those published before 2010. Two authors independently reviewed all abstracts and full-text articles to determine inclusion. In the case of disagreement, a third author acted as arbiter. Study evaluation consisted of grading level of outcomes using the Kirkpatrick model, assessing for the presence of key components of education innovation literature, and applying an author-driven global innovation rating. Among 3,830 identified publications, 350 (175 full texts and 175 abstracts) studies were selected for analysis. Only 13 studies were included from 2010 to 2011, with 98 from 2020 to 2021. The most common innovations were simulation (142), eLearning, including web-based software and video-based learning (78), 3-dimensional modeling/printing (34), virtual/augmented reality (26) podcasts/smartphone applications/social media (24), team-based learning (17), flipped classroom (17), problem-based learning (10), and gamification (9). Ninety-eight (28.0%) articles included a study design with a comparison group, but only 23 of those randomized learners to an intervention. Most studies relied on Kirkpatrick Level 1 and 2 outcomes—the perceptions of training by learners and acquisition of knowledge. The sustainability of the innovation, transferability of the innovation to a new context, and the explanation of the novel nature of the innovations were some of the least represented features. We rated most innovations as only slightly innovative. There has been an explosion of reports on educational methods in clinical neurology over the last decade, especially in simulation and eLearning. Unfortunately, most reports lack adequate assessment of the validity and effect of the respective innovation's merits, as well as details regarding sustainability and transferability to new contexts.

Neurology education is changing. Advances in adult learning theory and instructional technologies continue to present learners with new tools to acquire knowledge of key concepts. In a recent systematic review, data from 6,103 medical students and practitioners across varying education levels reported neurology as the most challenging specialty to learn, self-rated a lower level of knowledge, and reported a low level of confidence with the subject matter.^1^ These learners noted insufficient teaching in basic anatomy, neurologic physiology, and neurologic examination skills. They cited an overreliance on passive lectures, instead of bedside teaching and requested innovative methods to bridge the educational gap.^1^ Innovative approaches are being implemented to improve education and elevate learner confidence.

In recent years, many clinical neurology education innovations have been introduced; however, a current synopsis of these innovations is unavailable with the last review dating back nearly a decade.^2^ In the meantime, neurologic education research has accelerated. To better understand and elucidate the evolving landscape of neurology education, we conducted a scoping review to address the following research questions: (1) what novel education interventions are educators using in clinical neurology education? and (2) how innovative are the educational interventions?

## Methods

Given the dearth of randomized control trials designed to investigate interventions in neurology education and the lack of structured criteria for completing and reporting medical education research, we chose a scoping review design to include all measures of investigative work focused on improving neurology education delivery. The scoping review methodology provides opportunity to assess emerging evidence, investigate research conduct, identify knowledge gaps, and provide framework for future research development.^3-6^ We have not sought to evaluate the effectiveness or quality of education interventions as expected in a systematic review but instead methodically map the emerging literature of clinical neurology education. We followed Preferred Reporting Items for Systematic reviews and Meta-Analyses extension for Scoping Reviews (PRISMA-ScR) guidelines.^7^

### Eligibility Criteria

Inclusion criteria required the investigation of an educational innovation or intervention pertaining specifically to neurosciences. An education innovation was defined as any new education process, strategy, product, or approach aimed specifically at improving a current educational status. We included studies that assessed neurologic topics such as neuroanatomy, clinical neurology, and neurologic emergencies. Studies that focused on current or prior well-established education methods, including lecture-based didactics, were excluded. Study participants included medical students, residents, fellows, and attending physicians with studies pertaining to clinical specialties in neurology, pediatric neurology, and interventional neuroradiology. We excluded studies of premedical school learners and neurosurgical-focused studies.

### Information Sources and Search Strategy

A health sciences librarian developed the search strategy in consultation with the other authors. The following databases were searched on November 4, 2021: PubMed (PubMed.gov), Embase (Embase.com), Scopus (Scopus.com), Cochrane Library (WileyOnline; Cochrane Database of Systematic Reviews, Cochrane Central Register of Controlled Trials, Cochrane Methodology Register), Education Resources Information Center (EBSCOhost), and PsycINFO (EBSCOhost). The search strategy combined keywords and subject headings from the concepts of innovation, education, students, and neurology from 2010 through November 2021. Example search terms included virtual reality, instruction, and neurology. The eAppendix 1 (links.lww.com/NE9/A18) contains the full search strategies from each database.

### Study Selection and Data Collection

**Figure 1:**
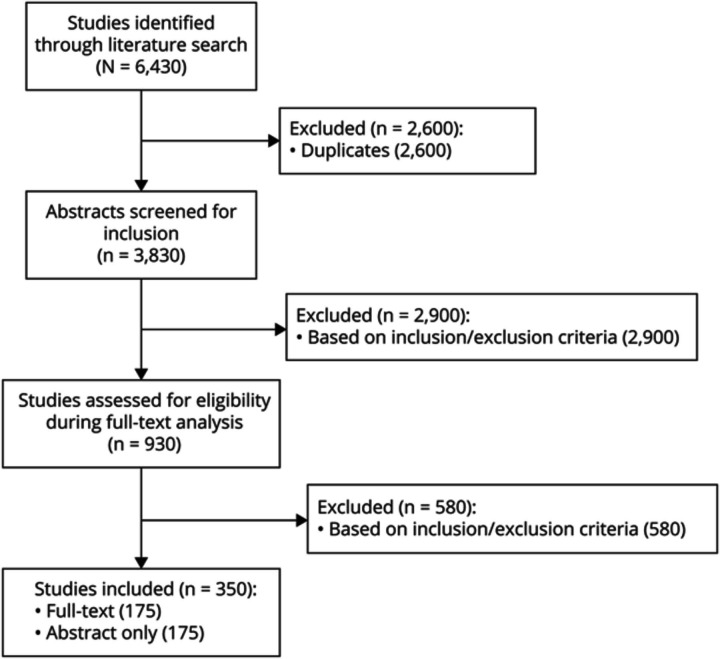
PRISMA-ScR Flowchart of Study Inclusion Flowchart of article inclusion phases. A total of 930 studies were included in full-text analysis and assessed by 2 screening authors, with 350 studies included in data extraction. PRISMA-ScR = Preferred Reporting Items for Systematic reviews and Meta-Analyses extension for Scoping Reviews.

Two authors (W.D.Z. and M.B.P.) independently reviewed all abstracts that met the criteria to determine advancement to full-text review. After the elimination of abstracts not meeting the inclusion criteria, 2 independent authors (W.D.Z. and M.B.P.) repeated full-text analysis to determine inclusion. In the case of disagreement, a third author (N.A.M.) acted as arbiter to determine final inclusion. Figure 1 demonstrates the PRISMA-ScR flow of article inclusion. Abstracts without corresponding full texts were included for completeness. One author (W.D.Z.) extracted authorship, publication year, study design, education innovation, neurologic topic, study participants, outcomes/evaluations, and results from each included study. Full extraction data of all studies are represented in eTable 1 (links.lww.com/NE9/A19).

### Study Evaluation

Study evaluation included assessment of the measured outcomes in each individual study and an audit of both the degree of innovation and the presence of key features that define an innovation article. We implemented the Kirkpatrick framework of training evaluation to assess the level of outcomes measured in both included full text and abstracts. Each study was scored on a scale of 1–4 based on the model. Level 1 demonstrated reaction, or the learner found the innovations relevant and engaging. Level 2 measured learning or whether the learner acquired the knowledge or skills predetermined by the designed intervention. Level 3 demonstrated behavior change as the learner applied reinforced actions to their training or clinical practice. Finally, Level 4 measured results or whether the innovation affected patient outcomes and clinical results.^8-12^ If a study did not include a prespecified outcome where the Kirkpatrick framework could be applied, the study was scored as not applicable.

**Figure 2:**
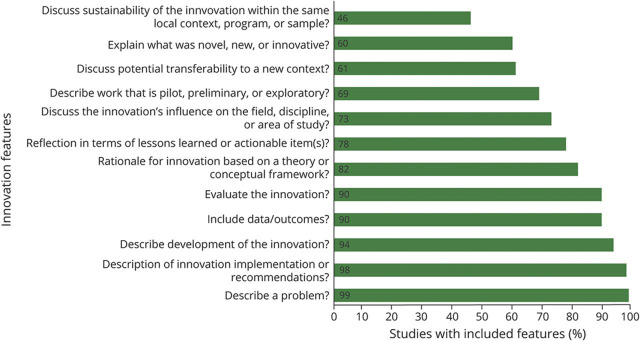
Defining Features of Education Innovation Audit of all 175 full-text studies for the presence of Colbert-Gert et al.^13^ defined 12 key features of education innovation articles, represented in percentages.

We assessed for key features of an innovation article according to the framework of Colbert-Getz et al.^13^ who recently defined these key features based on content analysis from author guidelines and publications describing what health professions education editors look for in innovation articles. The 12 key features determined in this systematic review can be seen in Figure 2, with a higher number of features more representative of an education innovation article. The scoring system does not actually rate how innovative a described innovation is, so Colbert-Betz et al. also incorporated a global innovation rating. They rated each innovation as not at all innovative, slightly innovative, or very innovative. One author (W.D.Z.) tabulated the key features for all full-text studies. Two authors (W.D.Z. and N.A.M.) replicated the global innovative rating process for all full-text studies. Interrater reliability was determined using weighted Cohen κ calculations.

### Data Availability

Anonymized data not published within this article will be made available by request from any qualified investigator.

## Results

**Figure 3:**
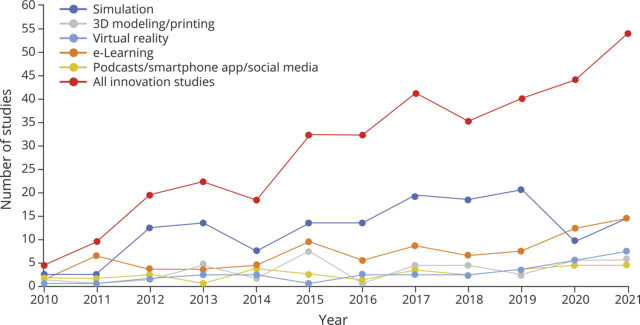
Top Education Innovation Study Trends Over Past Decade Most studied neurology educational innovations and their prevalence per year since 2010. A total of 4 studies published in 2010 included, with an increase to 54 published in 2021. Total studies included for each innovation: simulation (142 studies), eLearning (78 studies), 3D modeling/printing (34), virtual reality (26), and podcasts/smartphone app/social media (24). 3D = 3 dimensional.

Among 3,830 identified publications, 930 abstracts progressed to full-text analysis. Of those abstracts, 350 studies were selected for inclusion. One hundred seventy-five studies (50.0%) were full-text publications, and 175 (50.0%) abstract-only studies met the criteria (Figure 1). The most common innovations were simulation (142), eLearning, including web-based software and video-based learning (78), 3-dimensional (3D) modeling/printing (34), virtual reality (VR) or augmented reality (AR) (26), and podcasts/smartphone applications/social media (24). Other studied innovations included team-based learning (TBL) (17), flipped classroom (17), problem-based learning (PBL) (10), and gamification (9). The remaining modalities researched included art-driven learning and active learning. The proportion of the most frequently used types of innovation changed over the study period (Figure 3). Numerous studies investigated multiple education innovations simultaneously.

**Figure 4:**
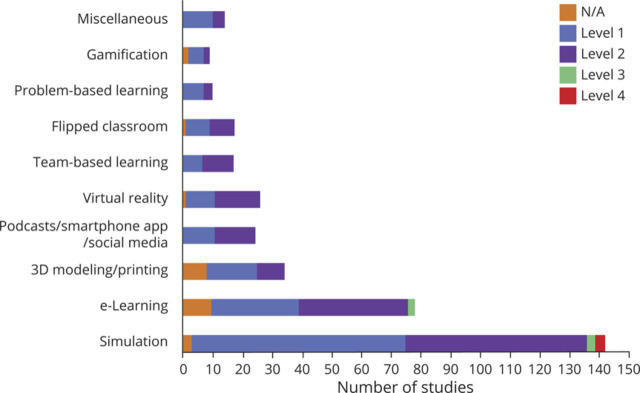
Kirkpatrick Training Evaluation Outcomes per Innovation Kirkpatrick training evaluation grades by level in total for each education innovation. There were 5 Level 3 studies and only 3 Level 4 studies. 3D = 3 dimensional; N/A = not applicable.

Ninety-eight (28.0%) articles included a comparison group for outcome analysis. The target audience was most often medical students and neurology residents. Most studies were classified as pilot studies, testing the feasibility of the innovation or concept; only 23 (6.6%) studies included a randomized study design. The number of studies published increased dramatically over time (Figure 3). Nearly half of all studies (171/350, 48.9%) met Kirkpatrick Level 1 evaluation. A substantial percentage (150/350, 42.9%) reported Level 2 outcomes. Only 5 studies (1.4%) reported Level 3 outcomes, and 3 (0.9%) studies reported Level 4 outcomes (Figure 4). A description of studies reporting Level 3 and 4 outcomes can be found in Table 1.^14-21^

**Table 1:** List of Studies With Level 3 or 4 on Kirkpatrick Framework Training Evaluation

Author	Study design	Education innovation	Comparison group	Outcomes	Results	Kirkpatrick assessment
Kelly et al.^14^	Investigation, interventional study	Simulation	N/A	Residents' self-perceived ability to effectively counsel patients and families before and after the exercise was compared. Trends in time to initiate IV thrombolysis for stroke were investigated before and after the intervention.	Residents' preparedness to perform counseling on acute stroke improved, though not significant (*p* > 0.05). Mean door-to-thrombolytic treatment for acute stroke was significantly improved in the 6 mo following simulation exercises compared with the previous 18 mo (54 vs 69 min, *p* = 0.01).	4
Mehta et al.^15^	Investigation, interventional study	Simulation	N/A	Resident performance pre- and post-intervention, critical action checklist completion, and door-to-needle time for IV-tPA.	Simulation training independently predicted reduction in door-to-needle time by 9.64 min (95% CI −15.28 to −4.01, *p* < 0.001) after controlling for age, night/day shift, work week vs weekend, and blood pressure at presentation.	4
Richardson et al.^16^	Interventional study	Simulation	Post- vs pre-simulation door-to-needle times	A mannequin was used and controlled by simulation laboratory personnel while participants were videotaped as they performed the scenario. Incorporated into the debriefing of the scenario was education of door-to-needle goals, stroke alert process, and a discussion of role expectations.	Postintervention, the door-to-needle average decreased to 39.8 min (*p* = 0.0794). Before the education, the goal of administering alteplase in less than 60 min occurred 80% of the time and following education the goal was met 89% of the time.	4
Dwortezky et al.^17^	Interventional, mixed-methods design	Simulation	N/A	Used a questionnaire assessing baseline knowledge and attitudes regarding seizure management. Calculated interobserver reliability of the checklist for consistency among the raters.	Training led to sustainable improvement in performance in the actual EMU. Knowledge in seizure management was significantly improved following the program. Interrater agreement was moderate to high.	3
Gordon et al.^18^	Interventional, comparative study	eLearning	Control facilities vs intervention facilities	The primary outcome variables were the percentage of residents receiving antipsychotic medications and the percentage who were physically restrained.	Residents in facilities were 75% less likely to be physically restrained and 17% less likely to be prescribed antipsychotic medication compared with residents in control facilities over the 18-mo intervention period (OR 0.25, *p* = 0.05) and (OR 0.83, *p* = 0.07)	3
Sivakumar et al.^19^	Interventional study	eLearning	N/A	Attainment of skill sets is tracked through self-reported case logs and professionalism. Resident and faculty surveys are conducted for feedback and system change.	Resident attendance and participation had significantly increased after the intervention.	3
Stork et al.^20^	Investigational, pilot study	Simulation	N/A	Entrance and exit surveys were administered to measure changes in knowledge, skills, and attitudes. The use of rtPA for AIS was tracked before and after the intervention.	All trainees reported significantly increased confidence in acute stroke patient selection and management with rtPA, including its potential complications. IV rtPA use increased by 75% in the 7 mo after workshop and increase persisted at 13 mo.	3
Zaika et al.^21^	Interventional, investigational study	3D modeling, simulation	N/A	Participants were assessed on their procedural pace, coiling quantity and quality, and perforation rates. Spatial ability was assessed using a mental rotations test and used in the performance analysis	Individuals were able to perform the procedure faster after 6 sessions, reducing their average time from 42 to 24 min. The coil success rate improved from 82% to 88%, and the coil packing rate remained consistent at 30% throughout testing.	3

Abbreviations: 3D = 3 dimensional; AIS = acute ischemic stroke; EMU = epilepsy monitoring unit; N/A = not applicable; OR = odds ratio; rtPA = recombinant tPA; tPA = tissue plasminogen activator.

First author, neuroscience topic, participants, comparison group in design, study outcomes, and results of the studies who met Kirkpatrick training evaluation Level 4 (3 studies) and Level 3 (5 studies) criteria.

We display the 12 defining features of innovation articles and their prevalence in Figure 2.^13^ The median (interquartile range) number of included features for the 175 full-text articles was 11 (10–12). The mean global innovation rating scored by author 1 (W.D.Z.) was 1.13 ± 0.70 and by author 2 (N.A.M.) was 0.98 ± 0.73. The 2 authors agreed on the same level of innovation for 90 of the 175 full-text publications (51.4%). The interrater reliability was noted to be fair (weighted κ = 0.23; 95% CI 0.12–0.35).^22,23^

### Innovations

#### Simulation Studies

##### Content and Design

**Figure 5:**
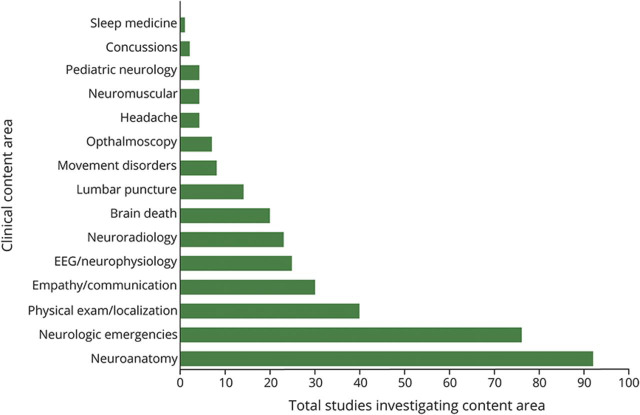
Clinical Content Areas (by Study) Total number of studies focused on each clinical content area represented in the entire 350 studies included in the scoping review.

Simulation was the most investigated education innovation, totaling ∼40% of included studies. Simulation displayed the widest range of content areas of all innovations, all within the realm of clinical neurology: diagnosis and management, procedural training, communication skills, and neuroanatomy/localization (Figure 5). Clinical neurology simulation studies (66.2%, 94/142 simulation studies) mainly focused on diagnostics and treatment of neurologic emergencies (70.2%, 66/94 studies), brain death testing (11.7%, 11/94 studies), or EEG interpretation (6.4%, 6/94 studies). Procedure-oriented simulation studies (17.6%, 25/142 studies) provided hands-on experience for beside procedures including lumbar puncture (10 studies), fundoscopy (4), and peripheral nerve blocks (3). Eight studies demonstrated the utility of simulation in interventional neuroradiology such as diagnostic angiography and aneurysmal coiling. Twenty studies focused on communication skills. Through simulation, study participants practiced disclosing devastating neurologic diagnoses, assisting the patient in adjusting to chronic disease, and explaining the concept of brain death. A minority of simulation studies (2.8%, 4/142 studies) focused on neuroanatomy and localization. Participants in the simulation studies ranged from medical students to attending physicians demonstrating the feasibility of learning across multiple levels of learners.

##### Evaluation

Most studies (50.7%, 72/142) evaluated participants' satisfaction as the primary outcome. Sixty-one studies (43.0%, 61/142 studies) assessed knowledge attainment through pre- and post-simulation testing, although few used a control group. Holling et al.^24^ found additional simulation-based education improved third-year medical students' performance in the clinical brain death examination compared with the traditional lecture format. Sun and Qi^25^ showed that neurology residents who participated in the study had a higher lumbar puncture success rate after completing the designated simulation and noted the experience to be more effective than traditional teaching methods. Three simulation studies reported Kirkpatrick Level 3 outcomes and focused on content areas of seizure management in the epilepsy monitoring unit, treatment of acute ischemic stroke (AIS), and procedural skills of coil deployment in aneurysm securement after subarachnoid hemorrhage.^17,20,21^ Three studies reported Kirkpatrick Level 4 outcomes, all of which demonstrated an improvement in the door-to-needle time for acute thrombolytic therapy for real patients following simulation training.^14-16^

#### eLearning Studies

##### Content and Design

**Figure 6:**
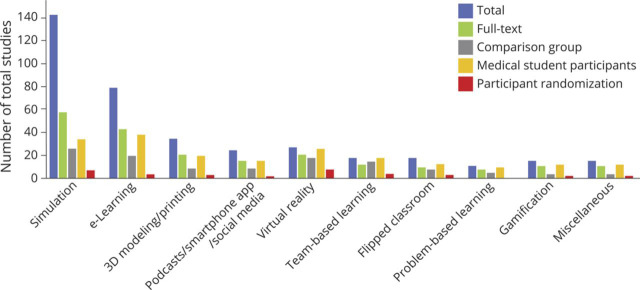
Study Design per Innovation Number of total studies with study design details including full-text publications, comparative group design, medical student participation, and participant randomization for each innovation. 3D = 3 dimensional.

eLearning studies encompassed online training modules, video lectures, web-based courses, computer-generated imaging, communication platforms, and cloud-based computing. Neurologic content areas included neuroanatomy (21/78 studies), clinical neurology (48/78 studies), neurologic emergencies (4/78 studies), and neuroradiology (5/78 studies). Specific clinical neurology concepts portrayed were neurophysiology (13 studies), neurologic localization (5 studies), movement disorders (4 studies), and neurophobia (2 studies). AIS was the most common neurologic emergency studied. Forty-two of the eLearning studies (53.8%) were full-text publications, with only 19 studies (24.4%) including a comparison group in the study design (Figure 6).

##### Evaluation

Twenty-nine and 37 studies reported Kirkpatrick Level 1 and 2 results, respectively. One Level 2 study had participants use a computer mouse to drag an embolus along the cerebrovascular tree to demonstrate clinical stroke syndromes based on the affected vessel and found stronger performance on posteducation testing in the interventional group.^26^ Ten studies did not have a measurable outcome, and only 2 studies reported Kirkpatrick Level 3 outcomes. Sivakumar et al. developed a novel web-based educational module inclusive of an online repository of instructional material, such as self-test tools and prerecorded lectures. Allowing for independent online learning, physician learners were able to follow and obtain predefined educational goals. The authors provided a pathway to combat time and schedule limitations for a busy resident schedule with significantly improved attendance and participation after intervention.^19^

#### 3D Modeling/Printing Studies

##### Content and Design

3D modeling or printing studies primarily focused on teaching neuroanatomy. Anatomic representations included a 3D printed cranioplasty skull model, a computer-generated imagery of pterygopalatine fossa, an atlas of cranial nerve pathology, a model of key anatomic structures involved in external ventricular drain (EVD) placement, and 3D video for skull base anatomy. Other content areas included neuroradiology (8.8%, 3/34 studies) and clinical neurology (8.8%, 3/34 studies). The target audience was primarily medical students. Only 8 (23.5%, 8/34 studies) of the 3D studies included a comparator.

##### Evaluation

In the 17 studies reporting Kirkpatrick Level 1 outcomes, participant consensus suggested enjoyment of 3D modeling or printing education. The 9 Kirkpatrick Level 2 studies all demonstrated significant participant knowledge acquisition. A study of 101 medical students randomized to either an experimental group building 3D color-coded physical models of the periventricular structures or a control group examining 2-dimensional brain cross-sections found that those who built the 3D models scored significantly higher on a postintervention neuroanatomy quiz.^27^ There was only 1 3D study that examined Kirkpatrick Level 3 and no Level 4 outcomes.

#### Podcasts/Smartphone Applications/Social Media Studies

##### Content and Design

Studies incorporating smartphones were grouped as podcasts, smartphone applications, or social media-oriented studies given the commonality of the delivery device. There were 24 total studies delivered via smartphone. Specifically, there were 5 podcasts (20.8%, 5/24 studies), 12 smartphone applications (50.0%, 12/24 studies), and 7 social media studies (29.2%, 7/24 studies). The podcasts contained clinical neurology material including neurophysiology, stroke, obstetrical neurology, and neuroradiology. Most podcast-focused studies were full text, with half targeting medical students and having a comparison group. Smartphone applications covered a wide range of clinical topics including a medication guide, localization tool, fundoscopy aid, and clinical description applications for common movement disorders and benign paroxysmal positional vertigo (BPPV). All but 3 of the smartphone application studies focused on clinical neurology. Social media-focused studies used Instagram, Twitter, Facebook, and Reddit as platforms. Three of these studies were full-text publications, and none of them used a comparative study design.

##### Evaluation

All but one of the podcast studies reported Kirkpatrick Level 2 outcomes. Seven smartphone application studies assessed participant subjective experience (Level 1), and 5 studies assessed knowledge acquisition (Level 2). In a randomized, prospective study, Dlugaiczyk et al. used an angular vestibulo-ocular reflex (aVOR) smartphone app to model the stimulation of the semicircular canals by rotational movement and the consequent patient eye movements. The authors randomized participants to small group instruction on therapeutic maneuvers for BPPV with or without the addition of training using the aVOR application. More participants in the aVOR group ordered the steps of the canalith repositioning procedure correctly.^28^ All 7 social media studies reported Kirkpatrick Level 1 outcomes, demonstrating participant satisfaction.

#### VR/AR Studies

##### Content and Design

More VR/AR studies (69.2%, 18/26 studies) progressed to full-text publications than other types of studies. Most (57.7%, 15/26 studies) of them taught neuroanatomy. Eleven studies (42.3%) investigated clinical neurology topics such as cranial nerve palsies, EEG interpretation, EVD placement and management, and the neurologic examination. Nearly all studies targeted medical students. Of the VR studies, 17/26 studies (65.4%) contained a comparator, with the control group tending to be a lecture-based education. From the 23 randomized control trials, 8 specifically focused on VR instructional methods.

##### Evaluation

Participants from all studies were satisfied with the VR/AR education. The majority of VR/AR studies assessed Kirkpatrick Level 2 outcomes. A study of 66 preclinical medical students randomized to either VR- or paper-based instruction of neuroanatomic structural relationships did not find a difference in postintervention test scores. However, those that received VR instruction did demonstrate subjective decline in neurophobia.^29^ Every VR/AR study assessing Kirkpatrick Level 2 outcomes demonstrated sufficient knowledge transfer, with 14/15 studies showing statistically significant improvement after the VR intervention either in pre- and post-test or interventional vs control group comparisons.

#### TBL Studies

##### Content and Design

Seventeen (4.9%) studies used a TBL strategy as their education innovation, implementing a modular instructional approach based on prepreparation, in-class readiness assurance testing, and application-focused exercises. TBL studies most commonly taught clinical neurology (94.1%, 16/17 studies) including but not limited to the evaluation of altered mental status, polyneuropathies, behavioral neurology, geriatric medicine, and neuroimaging. A significant percentage of TBL studies were full text (70.6%, 12/17 studies) and included a design with a comparison group (82.4%, 14/17 studies). All 17 studies targeted medical students, with the comparison groups in these studies being a traditional didactic, lecture-based curriculum.

##### Evaluation

Kirkpatrick levels assessed were Level 1 in 7 studies and Level 2 in 10 studies, with no Level 3 or 4 outcomes. Most participants enjoyed TBL. All Kirkpatrick Level 2 studies demonstrated positive results of knowledge acquisition on postintervention evaluations. One specific Level 2 study showed that participants who experienced the TBL innovation scored higher than participants before the intervention and were more likely to receive a higher grade (A vs B or C) for the educational block than the previous block.^30^

#### Flipped Classroom Studies

##### Content and Design

Over half of the studies evaluating flipped classrooms (52.9%, 9/17 studies) were published as full texts. Medical students nearly always comprised the target audience. Seven studies included a comparison group, described as traditional didactic instruction. In 2 of the 7 studies with a comparator, the learners were randomized. Content areas included either neuroanatomy (47.1%, 8/17 studies) or clinical neurology (52.9%, 9/17 studies), with topics including physical examination skills, EEG fundamentals, and the concept of coma.

##### Evaluation

One study did not report any outcomes; 8 studies reported Kirkpatrick Level 1 outcomes, and 8 reported Kirkpatrick Level 2 outcomes. One of the 2 randomized studies investigating a flipped classroom intervention to teach neuroanatomy found participants perceived educational resources as very useful. However, although the flipped classroom innovation increased knowledge postintervention, it was not superior to traditional didactic lectures.^31^ In the other randomized study, the authors used the flipped classroom technique to teach typical EEG patterns. They demonstrated significant improvement in posttest scores in the flipped classroom group after an active EEG interpretation module was completed compared with those who received no prelecture material or instruction.^32^

#### PBL Studies

##### Content and Design

PBL studies investigated a wide range of clinical neurology topics including concussion physiology, neurologic examination skills, and neurologic case presentations. Nine of the 10 studies (90.0%) targeted medical students, and only 4 (40.0%, 4/10 studies) used a comparison group.

##### Evaluation

Seven studies (70.0%) reported Kirkpatrick Level 1 outcomes, and 3 studies (30.0%) reported Level 2 outcomes. Several PBL studies aimed at combating neurophobia by building confidence through individual neurologic cases. Wiznia et al.^33^ demonstrated that after a PBL innovation, a group of 100 medical students felt that the interaction among students increased and most of the participants preferred the PBL format, leading to increased learning. Overall, the studies found PBL to be a useful adjunct to traditional instruction models.

#### Gamification Studies

##### Content and Design

Nine gamification studies (presented as 7 abstracts and 2 full-text articles) described jeopardy-based trivia, card games, roleplay, board games, and television-themed games. The games required multiple-choice responses, free responses, anatomic drawings, or group problem-solving to answer questions regarding neuroanatomy, neuroradiology, neurology semiology, and movement disorders. Only a single study in this group had a comparison group.

##### Evaluation

Five of the gamification studies assessed Kirkpatrick Level 1 outcomes, and 2 assessed Level 2 outcomes. Most of the studies demonstrated participant satisfaction. A study comparing examination scores between 43 medical students who engaged in a jeopardy-based game to 41 historic controls found no difference in the postclerkship shelf examination scores between the 2 groups. Participant feedback was mixed, with only 41.2% finding the intervention at least moderately helpful.^34^ In contrast, a neurology-based, interactive, card game provided a highly enjoyable new teaching method for neurologic semiology as rated by 112 medical students and demonstrated statistically significant improvement in a multiple-choice questionnaire postintervention.^35^ There were no studies with Level 3 or 4 outcomes.

#### Miscellaneous Studies

##### Content and Design

Other innovations included art-driven education and active learning. Most art-oriented studies (71.4%, 5/7 studies) highlighted neuroanatomic concepts and targeted medical students. Participants either drew or clay-modeled spinal cord anatomy or periventricular structures of the brain. Seven studies used active learning as an educational innovation in the study design. Five of these studies incorporated roleplay to teach clinical neurology topics including communication skills and brain death. Active learning was used in only 2 full-text studies, with most of these studies using medical student participants. Only 1 study compared outcomes with controls.

##### Evaluation

Five art-oriented studies assessed Kirkpatrick Level 1 outcomes, with all reporting participant satisfaction. One study focused on brain imaging interpretation randomized clinical medical students to either drawing CT or MRI pathology such as a subdural hematoma or standard, lecture-based teaching. Although preintervention evaluation scores showed no difference in the 2 groups, the drawing group scored higher on a final evaluation.^36^ Five active learning studies assessed Kirkpatrick Level 1 outcomes, and 2 studies assessed Level 2. By having neurology residents or medical students physically act out neurologic deficits, these studies showed that neurophobia was decreased and participants preferred this teaching method over the traditional didactic lecture style.^37^ All active learning studies demonstrated participant satisfaction. In 1 study, the authors used a hands-on ultrasound workshop that included transcranial Doppler ultrasound, ocular ultrasound, ultrasound-guided EVD placement, high-intensity focused ultrasound for brain lesions, carotid artery scan with ultrasound, and ultrasound-guided central line placement to teach key anatomic concepts. The posttest scores were significantly higher after the intervention and provided exposure to key neuroanatomic concepts such as cerebral vasculature, neck anatomy, CSF flow, and optic nerve appearance.^38^

### Coronavirus Disease 2019

Nine studies specifically discussed the viral pandemic and its effect on neurologic education innovations. Most (77.8%, 7/9 studies) studies focused on eLearning as a remote learning solution. One study focused on neuroanatomy, with the remaining studies focused on clinical neurology topics. Some of the eLearning study designs included interactive case discussions and independent films portraying neurologic syndromes. These studies primarily used medical students as participants, with only 2 studies including a comparison group. Eighty-nine percent of the studies reported Kirkpatrick Level 2 outcomes. Multiple studies demonstrated a high level of learner satisfaction. Some participants stated that the eLearning innovations during the early pandemic isolation facilitated access to and strong communication with the course instructor.

## Discussion

This scoping review identified numerous innovations in neurology education including simulation, eLearning, 3D modeling/printing, VR, and podcasts/smartphone/social media, among others. Reporting of new educational modalities increased drastically over time. We included abstract-only and full-text articles to demonstrate the wide range of ingenuity and imagination educators have applied to fostering educational growth, even if the delivery tool was in its infancy and had not reached formal publication. Innovations included clay modeling of the spinal cord, 3D printing of the pterygopalatine fossa, and an immersive VR educational experience.^29,39,40^ Without inclusion of the abstract-only articles, we would have failed to demonstrate several interventions that may warrant future attention.

A similar review written in 2013 highlighted a lack of high-level evidence to support specific teaching methods.^2^ Our review similarly revealed a dearth of rigorously designed randomized controlled trials with relevant high-level Kirkpatrick outcomes that allow for proper assessment of studied innovations. Indeed, most studies only reported participant satisfaction; however, it is uncertain whether participant satisfaction represents a valid outcome measure.^41^ In fact, studies suggest that learners may express dissatisfaction toward more active teaching styles that achieve better educational outcomes.^42^ We similarly revealed a dearth of studies implementing rigorous qualitative methodology.

The neurology education researcher or educator must continue to strive to determine which teaching methods are best for specific topics, learner populations, and learning environments. Merit of an educational innovation would be best tested against traditional teaching formats and other innovative methods. We found head-to-head comparisons of innovative teaching methods are rare, likely due to the difficulty in conducting such trials due to time, staffing, and financial constraints. Although few studies in our review used a randomized design, a growing number included a control group and at least reported outcomes of knowledge acquisition suggesting gradual improvements in methodological rigor and study quality, increasing the yield and influence on the field.

Our audit of key components that encompass an education innovation article showed large gaps in reporting sustainability and transferability across learner levels and content areas. As others have described, we found it difficult to concisely summarize reports owing to the many different shapes an innovation article could take, including departure from the classic design of introduction, methods, results, and discussion.^13^ Our interrater reliability was noted only to be fair when assessing the level of innovation, likely due to differing degrees of experience with each innovation, training backgrounds, and personal educational goals. Creating a more standardized construct of what an education innovation encompasses, what makes it novel, and how to prove sustainability must be a primary goal for future neurology education researchers.

The most represented innovation was simulation. Medical simulation facilitates assessment of higher-level outcomes such as behavioral change, lacking from assessments of other innovations. Several simulation studies demonstrated improved door-to-needle time in the management of patients with AIS.^14-16^ Although neurology simulation has made great strides, it has been limited in content areas. Future simulation studies can consider incorporating unrepresented subspecialty material such as movement disorders, neuromuscular diseases, and neuroimmunology (Figure 5). Furthermore, although some content areas such as procedural skills are well suited to stimulation techniques, others, such as neuroanatomy, are likely better taught through alternative modalities. More work is needed to decipher the optimal method to be implemented by the educator for each content area.

Our review revealed a predilection for innovation in neurology educational initiatives targeting medical students (Figure 6). One significant issue with investigating this learner level is the difficulty in evaluating the innovation's effect on patient outcomes. Focusing on graduate medical education of neurology trainees, including residents and fellows, who are consistently responsible for the care of patients, is an easier target for future studies to bridge the gap and demonstrate higher-level outcomes. Despite impressive progress in neurology education, our search, which resulted in the exclusion of many studies focused on neurosurgical education, emphasized that much remains to be done. Indeed, a recent scoping review in neurosurgery education included 533 full-text studies compared with the 175 that we discovered.^43^

Core journals in clinical neurology have rarely published articles that focus on education, due to a lack of submissions, lack of scientific rigor in candidate manuscripts, or absence of clarity on the role of education innovation. In comparison, other specialties, like general surgery have several journals dedicated to advancing education in their field including *the Journal of Surgical Education*, *Annals of Surgical Education*, and *Global Surgical Education*. In addition, many studies dedicated to neurology education have not been featured in general medical education journals. We predict that with an increase in the number of journals focused on medical and specifically neurology education and new reporting guidelines such as Defined Criteria To Report INnovations in Education (DoCTRINE), the quantity and quality of publications will continue to multiply and improve exponentially.^44^

Our review revealed significant variations in study design and reporting in recent neurology medical education literature. In the medical research literature, numerous reporting guidelines are followed, such as Consolidated Standards of Reporting Trials for randomized, controlled trials or PRISMA for systematic reviews.^45,46^ The Standards for Quality Improvement Reporting Excellence in Education (SQUIRE-EDU) was developed in 2016 to improve quality of reporting health professionals' education research through notation of educational gaps, documentation of the dependability of the educational intervention, and consideration of the effect of education improvement on the health care system.^47^ Despite these guidelines, few studies in this review noted application of this tool in the methods section. Given the increase in acceptance of the contributions of qualitative educational research, utilization of these guidelines must increase to maximize applicability and implementation.

Our scoping review has limitations. Although we investigated and organized a vast literature to highlight educational innovations in neurology, we did not complete a systematic review; therefore, we were not able to comment on a change in practice or scope but instead provided framework of existing interventions that can serve as scaffold for future investigations. Because of the scoping review design, we did not seek to grade the quality of the evidence (as opposed to the level of outcomes and inclusion of innovative features). For similar reasons, we cannot quantitatively or qualitatively answer questions regarding superiority of individual education innovations over traditional didactic teaching or contributory merits of furthering educational growth. Because of the lack of standardization in the medical education innovation literature, it is impossible to exclude sampling bias during article selection by the authors.

## Conclusions

Innovations in neurology education are increasing over time and are progressively reporting higher-level outcomes. Learners now have access to on-demand eLearning and podcasts, as well as opportunities to cement neuroanatomical relationships though 3D formats (3D printed models, 3D computer-rendered models, and VR). Educators have options for delivering content through a variety of formats that promote learning including flipped classrooms, TBL, and simulation. Education researchers, meanwhile, can continue to improve study design methods, outcome assessments, and reporting through the implementation of guidelines such as SQUIRE-EDU and DoCTRINE to improve the merits of educational studies. The goal is for neurology educators to bridge the gap from the learning environment to the patient's bedside with measurable effects on clinical outcomes.

## Appendix Authors

**Table:**

Name	Location	Contribution
William Denney Zimmerman, DO	Department of Neurology, University of Maryland School of Medicine; Program in Trauma, R Adams Cowley Shock Trauma Hospital, Baltimore, MD	Drafting/revision of the manuscript for content, including medical writing for content; major role in the acquisition of data; study concept or design; and analysis or interpretation of data
Melissa B. Pergakis, MD	Department of Neurology, University of Maryland School of Medicine; Program in Trauma, R Adams Cowley Shock Trauma Hospital, Baltimore, MD	Drafting/revision of the manuscript for content, including medical writing for content; major role in the acquisition of data; study concept or design; and analysis or interpretation of data
Emily F. Gorman, MLIS, AHIP	Health Sciences and Human Services Library, University of Maryland, Baltimore	Major role in the acquisition of data and study concept or design
Melissa Motta, MD	Department of Neurology, University of Maryland School of Medicine; Program in Trauma, R Adams Cowley Shock Trauma Hospital, Baltimore, MD	Drafting/revision of the manuscript for content, including medical writing for content, and analysis or interpretation of data
Peter H. Jin, MD	Department of Neurology, University of Maryland School of Medicine, Baltimore	Drafting/revision of the manuscript for content, including medical writing for content, and analysis or interpretation of data
Rachel Marie E. Salas, MD, MEd	Department of Neurology, Johns Hopkins School of Medicine, Baltimore, MD	Drafting/revision of the manuscript for content, including medical writing for content
Nicholas A. Morris, MD	Department of Neurology, University of Maryland School of Medicine; Program in Trauma, R Adams Cowley Shock Trauma Hospital, Baltimore, MD	Drafting/revision of the manuscript for content, including medical writing for content; major role in the acquisition of data; study concept or design; and analysis or interpretation of data

## Glossary

3D: 3 dimensional
AIS: acute ischemic stroke
AR: augmented reality
aVOR: angular vestibulo-ocular reflex
BPPV: benign paroxysmal positional vertigo
DoCTRINE: Defined Criteria To Report INnovations in Education
EVD: external ventricular drain
PBL: problem-based learning
SQUIRE-EDU: Standards for Quality Improvement Reporting Excellence in Education
TBL: team-based learning
VR: virtual reality
